# Re-annotation of the woodland strawberry (*Fragaria vesca*) genome

**DOI:** 10.1186/s12864-015-1221-1

**Published:** 2015-01-27

**Authors:** Omar Darwish, Rachel Shahan, Zhongchi Liu, Janet P Slovin, Nadim W Alkharouf

**Affiliations:** Department of Computer and Information Sciences, Towson University, 7800 York Road, Towson, Maryland 21252 USA; Department of Cell Biology and Molecular Genetics, 0229 Biological Science Research Building, University of Maryland, College Park, Maryland 20742 USA; USDA/ARS Genetic Improvement of Fruits and Vegetables Laboratory, BARC-W 10300 Baltimore Ave, Beltsville, Maryland 20705 USA

**Keywords:** Annotation, Strawberry, *Fragaria vesca*, Transcriptome, Genome, RNA-Seq, Gene, Rosaceae

## Abstract

**Background:**

*Fragaria vesca* is a low-growing, small-fruited diploid strawberry species commonly called woodland strawberry. It is native to temperate regions of Eurasia and North America and while it produces edible fruits, it is most highly useful as an experimental perennial plant system that can serve as a model for the agriculturally important *Rosaceae* family. A draft of the *F. vesca* genome sequence was published in 2011 [Nat Genet 43:223,2011]. The first generation annotation (version 1.1) were developed using GeneMark-ES+[Nuc Acids Res 33:6494,2005]which is a self-training gene prediction tool that relies primarily on the combination of *ab initio* predictions with mapping high confidence ESTs in addition to mapping gene deserts from transposable elements. Based on over 25 different tissue transcriptomes, we have revised the *F. vesca* genome annotation, thereby providing several improvements over version 1.1.

**Results:**

The new annotation, which was achieved using Maker, describes many more predicted protein coding genes compared to the GeneMark generated annotation that is currently hosted at the Genome Database for *Rosaceae* (http://www.rosaceae.org/). Our new annotation also results in an increase in the overall total coding length, and the number of coding regions found. The total number of gene predictions that do not overlap with the previous annotations is 2286, most of which were found to be homologous to other plant genes. We have experimentally verified one of the new gene model predictions to validate our results.

**Conclusions:**

Using the RNA-Seq transcriptome sequences from 25 diverse tissue types, the re-annotation pipeline improved existing annotations by increasing the annotation accuracy based on extensive transcriptome data. It uncovered new genes, added exons to current genes, and extended or merged exons. This complete genome re-annotation will significantly benefit functional genomic studies of the strawberry and other members of the *Rosaceae*.

## Background

The diploid strawberry *Fragaria vesca* is native to temperate regions of Eurasia and North America and is commonly known as the alpine or woodland strawberry. Due to its small size and small genome it is a versatile experimental perennial plant system and an emerging model for the *Rosaceae* family. The extant genome exhibits synteny with other commercially important members of the *Rosaceae* family such as apple (*Malus domestica*) and peach (*Prunus persica*) [1] and an ancestral *F. vesca* genome contributed to the genome of the octoploid dessert strawberry (*F.* × *ananassa*). Information obtained from studies of all aspects of plant growth, biochemistry, and physiology of *F. vesca* should be applicable to or inform studies of other *Rosaceae* species [1].

The time, cost, and difficulty of generating transcriptome sequences has been greatly reduced due to recent advances in sequencing technology, and RNA-Seq is now dominant over microarrays for in-depth transcriptome studies. The Illumina HiSeq 2000 platform was previously used to sequence 50 RNA-Seq libraries of 25 different *F. vesca* tissue types from early developing fruit at various stages, young leaves, and seedlings [2] of the 7^th^ generation inbred line Yellow Wonder 5AF7 (YW5AF7) [3]. The 50 libraries represent two biological replicates of 25 tissue types, and each library yielded between 12 and 40 million 51 bp, single end reads, for a total of ~70 Giga bytes of sequence data [2,4].

The genome sequence of the *F. vesca* inbred line Hawaii4×4 was published in 2011 [5] and the first version of the gene predictions is hosted at the Genome Database for *Rosaceae* (GDR) http://www.rosaceae.org/projects/strawberry_genome/v1.1/assembly [4]. In 2013, The National Center for Biotechnology Information (NCBI) published a new *F. vesca* annotation using the NCBI eukaryotic gene prediction tool Gnomon. Both annotations, from the GDR and the NCBI, are based on *ab initio* gene predictions and alignment of high confidence ESTs.

Using Bowtie2 [6] with default parameters, an average of 80.32% of the transcriptome reads from each library aligned to the genome (version 1.1), while only an average of 60.58% of these sequence reads aligned to the current gene predictions at GDR. Visualization of the mapped reads using GBrowse [7] uncovered incidences of genes of incorrect size, mis-annotated intron/exon junctions, and reads mapping to the genome that could represent non-coding transcripts, indicating that current annotation would be improved by incorporating the RNA-Seq data.

For the new annotation we used the MAKER2 annotation pipeline [8,9] to combine the following data sources: 1) *de novo* and reference based assemblies of the 50 RNA-Seq transcriptomes, 2) RefSeq alignments of publicly available plant transcripts, 3) current annotations from GDR, and 4) *ab initio* gene predictions based on analysis by SNAP, Augustus and GeneMark [10-13].

The resulting *F. vesca* genome re-annotation increases the number of coding regions and the total coding length across all seven linkage groups (LG1 - LG7) and the non-anchored scaffolds (LG0). This increase is due to the addition of exons to existing genes, the extension or merging of current exons, correction of intron/exon junctions, and the discovery of additional genes. Overall, this new annotation, named TowU_Fve, provides an improved annotation file and facilitates future gene isolation and identification in strawberry and other *Rosaceae* species.

## Results and discussion

### *De novo* transcriptome generation and assembly

**Figure 1:**
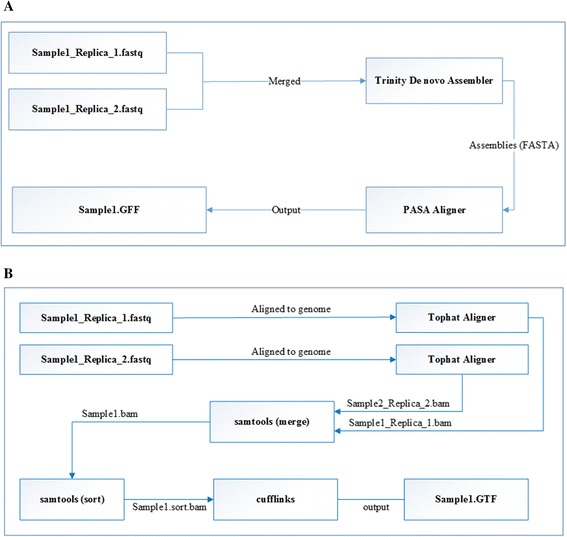
**Diagram illustrating two alternative assembly pipelines. (A)** *De novo* assembly and alignment pipeline using Trinity and PASA. **(B)** Reference guided assembly pipeline using TopHat and Cufflinks.

The *de novo* assembly pipeline shown in (Figure 1A) was used to assemble the reads from 50 stage and tissue libraries, resulting in 754,400 transcripts. The average number of assembled transcripts across different samples is 30,176 (Table 1), with the minimum number of assembled transcripts found in the early stage embryo (Embryo3) and the maximum number found in the leaf (Leaf1). All *de novo* assembled transcripts were aligned to the *F. vesca* genome using GMAP [14] within PASA, with the aims of eliminating sequences not aligning to the genome and merging *de novo* assembled sequences to remove redundancy. An average of 88.32% of the *de novo* assembled transcripts from each sample aligned to the genome.

**Table 1:** **Statistical summary of the de novo and reference based assembly results**

Sample ID*	**Number of raw reads**	**Reference-guided assembled transcripts**	***De novo*** **assembled transcripts**	**PASA alignment**
Cortex1	28,688,674	54,198	33,909	29,533
Cortex2	34,602,978	51,586	29,001	25,339
Cortex3	29,332,631	49,470	26,746	23,988
Cortex4	30,473,993	49,434	26,909	23,799
Cortex5	27,773,042	49,387	26,249	23,456
Embryo3	13,580,328	46,827	21,362	19,547
Embryo4	22,817,240	52,203	28,728	25,371
Embryo5	20,596,516	50,390	26,655	24,038
Ghost3	20,131,210	53,322	31,702	28,050
Ghost4	24,729,472	54,355	33,985	29,612
Ghost5	21,893,808	52,645	30,382	27,037
Ovule1	28,983,033	53,794	34,796	30,060
Seed2	31,044,314	54,353	31,669	28,044
Pith1	26,828,710	53,968	33,015	28,971
Pith2	29,684,268	51,985	28,916	25,497
Pith3	32,039,484	50,854	28,047	24,757
Pith4	31,461,556	50,342	26,944	23,872
Pith5	35,919,060	51,016	27,727	24,411
Wall1	25,996,860	53,102	31,968	28,214
Wall2	29,527,238	53,552	32,761	28,780
Wall3	19,130,041	51,998	30,787	27,384
Wall4	27,737,593	52,606	31,071	27,423
Wall5	34,216,551	52,882	32,980	28,694
Leaf1	30,740,916	54,993	36,502	31,610
Seedling1	27,518,958	53,477	31,589	28,065
Total	685,448,474	1,302,739	754,400	665,552

*Sample name indicates tissue type and the number indicates stage (see Kang et al. [2]). Each sample reflects averaged data from two biological replicates. Sample descriptions are available at http://bioinformatics.towson.edu/strawberry/newpage/Tissue_Description.aspx.

### Reference based assembly

Next, we carried out reference-guided assembly using TopHat (http://ccb.jhu.edu/software/tophat/) and Cufflinks (http://cole-trapnell-lab.github.io/cufflinks/) (Figure 1B). TopHat aligned RNA-Seq reads to the reference genome and identified exon-exon splice junctions. Cufflinks then used the alignment generated by TopHat and GeneMark gene models to assemble a total of 1,302,739 reference based transcripts, with the average being 52,110 and the minimum and maximum number found in the same tissues as for the *de novo* assembly (Table 1).

### *F. vesca* genome re-annotation pipeline

**Figure 2:**
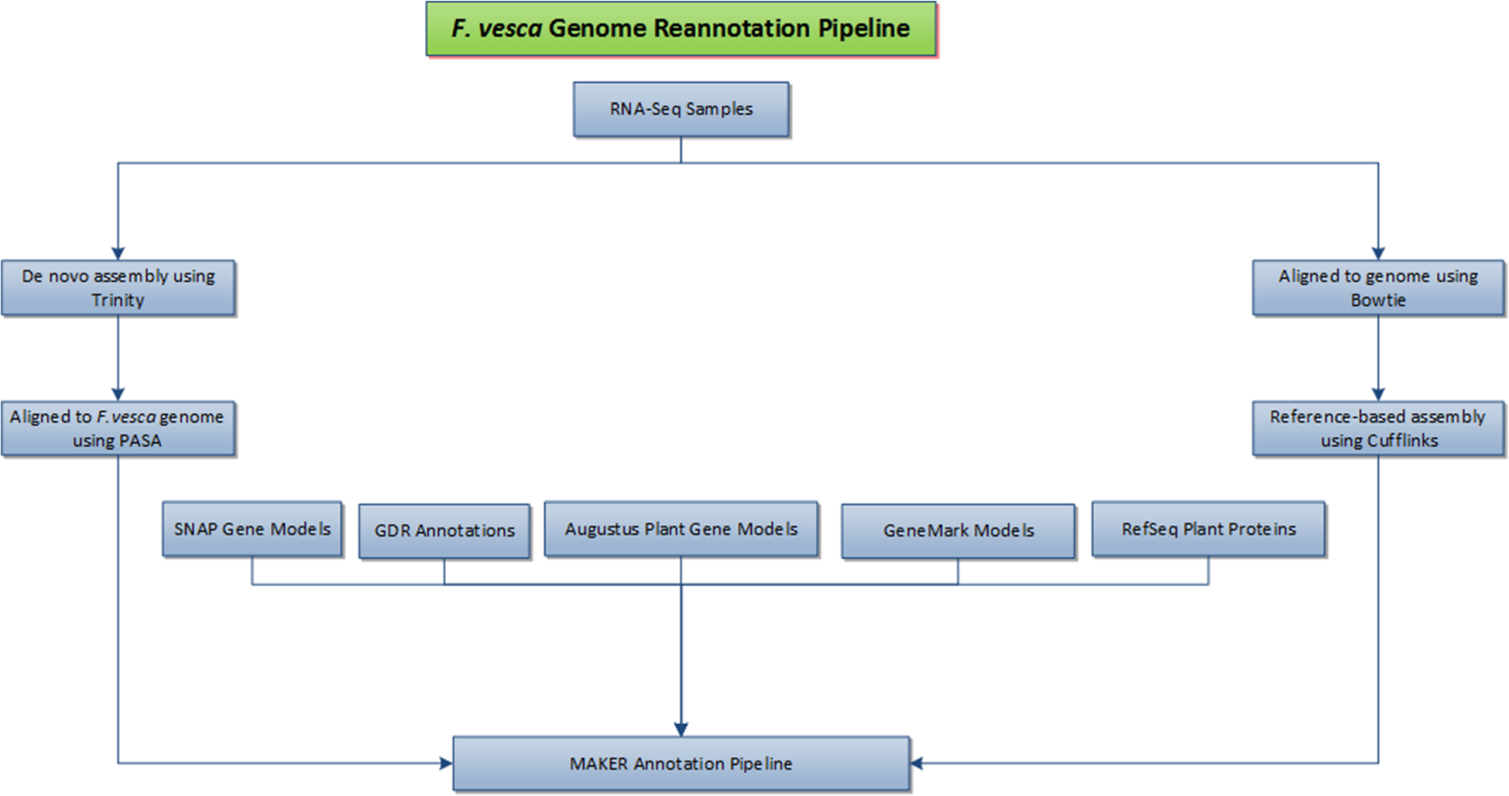
**Summary of overall bioinformatics pipeline for** ***F. vesca*** **genome re-annotation.** Evidence data including de novo assembled transcripts from all 50 samples, reference-guided assembled transcripts from all 50 samples, gene models generated using *ab initio* algorithm based tools (Augustus, SNAP and GeneMark), the first generation *F. vesca* gene predictions and plant reference proteins were passed to the MAKER pipeline to generate TowU_Fve annotation.

We then used the MAKER annotation pipeline (Figure 2) to generate the revised *F. vesca* annotation. MAKER is able to generate *ab initio* gene prediction using several tools within its pipeline; it identifies repeats, aligns proteins and ESTs to a genome, and automatically combines all classes of evidence data into gene annotations. Data analyzed in the MAKER pipeline included: 1) 754,400 *de novo* assembled transcripts from 25 samples (Table 1), each with two biological replicates; 2) trained *ab initio* predictions from the SNAP gene prediction tool; 3) Augustus trained datasets of *Arabidopsis thaliana* and *Solanum lycopersicum* (tomato) transcriptomes; 4) first generation *F. vesca* gene predictions obtained from the Genome Database for *Rosaceae*; 5) reference-based assemblies (1,302,739) obtained by aligning all RNA-Seq samples to the *F. vesca* genome version 1.1 using Cufflinks; and 6) plant reference proteins downloaded from the Universal Protein Resource (UNIPROT) database. This second-generation annotation for *F. vesca* is named TowU_Fve and is available at GDR (http://www.rosaceae.org).

### Comparison of TowU_Fve annotation with the prior annotation (version 1.1)

The TowU_Fve annotation increased the number of coding regions by 9,139 compared to the version 1.1 annotation. This translates into over two million base pairs of extra coding DNA sequence (CDS) (Table 2).

**Table 2:** **Statistical comparisons between first generation annotation and TowU_Fve annotation**

**Chr.**	**Annotation source**	**Gene count**	**CDS count**	**Total coding length (bp)**	**Avg. CDS length (bp)**
**LG0**	Version 1.1 Annotation	1,618	7,637	1,779,011	232.95
	TowU_Fve Annotation	1,729	8,172	1,887,397	230.96
**LG1**	Version 1.1 Annotation	3,440	17,714	4,064,386	229.44
	TowU_Fve Annotation	3,367	18,654	4,232,525	226.9
**LG2**	Version 1.1 Annotation	4,051	21,502	4,850,631	225.59
	TowU_Fve Annotation	4,140	22,637	5,076,584	224.26
**LG3**	Version 1.1 Annotation	4,920	24,334	5,804,654	238.54
	TowU_Fve Annotation	5,054	25,892	6,259,234	241.74
**LG4**	Version 1.1 Annotation	3,837	19,431	4,369,641	224.88
	TowU_Fve Annotation	3,956	20,445	4,568,144	223.44
**LG5**	Version 1.1 Annotation	4,655	23,920	5,587,403	233.59
	TowU_Fve Annotation	4,882	25,609	5,944,185	232.11
**LG6**	Version 1.1 Annotation	6,453	32,868	7,651,854	232.81
	TowU_Fve Annotation	6,547	34,777	8,067,688	231.98
**LG7**	Version 1.1 Annotation	3,857	19,864	4,642,955	233.74
	TowU_Fve Annotation	3,821	20,223	4,780,057	236.37
**Total**	Version 1.1 Annotation	**32,831**	**167,270**	**38,750,535**	**231.44**
	TowU_Fve Annotation	**33,496**	**176,409**	**40,815,814**	**231**

As summarized in Table 3, there are 2,286 newly predicted gene models (genes models that do not overlap with any of the genes from version1.1 annotation) in TowU_Fve. The number of newly identified coding exons is 6,006, and the average length of each of these exons is 183bp. The total coding length in all 7 linkage groups was found to be over 1.1 Mb.

**Table 3:** **Statistical summary of the newly predicted gene models by TowU_Fve annotation**

**Pseudo-chromosome**	**# of gene models**	**# CDS**	**Total coding length (bp)**	**Avg. CDS length (bp)**
**LG0**	111	268	49,025	182.93
**LG1**	78	247	39,746	160.92
**LG2**	262	670	113,528	169.45
**LG3**	345	794	144,502	181.99
**LG4**	247	596	105,811	177.54
**LG5**	301	703	123,173	175.21
**LG6**	412	930	158,501	170.43
**LG7**	530	1,798	442,946	246.36
**Total**	**2286**	**6006**	**1177232**	**183.10**

**Figure 3:**
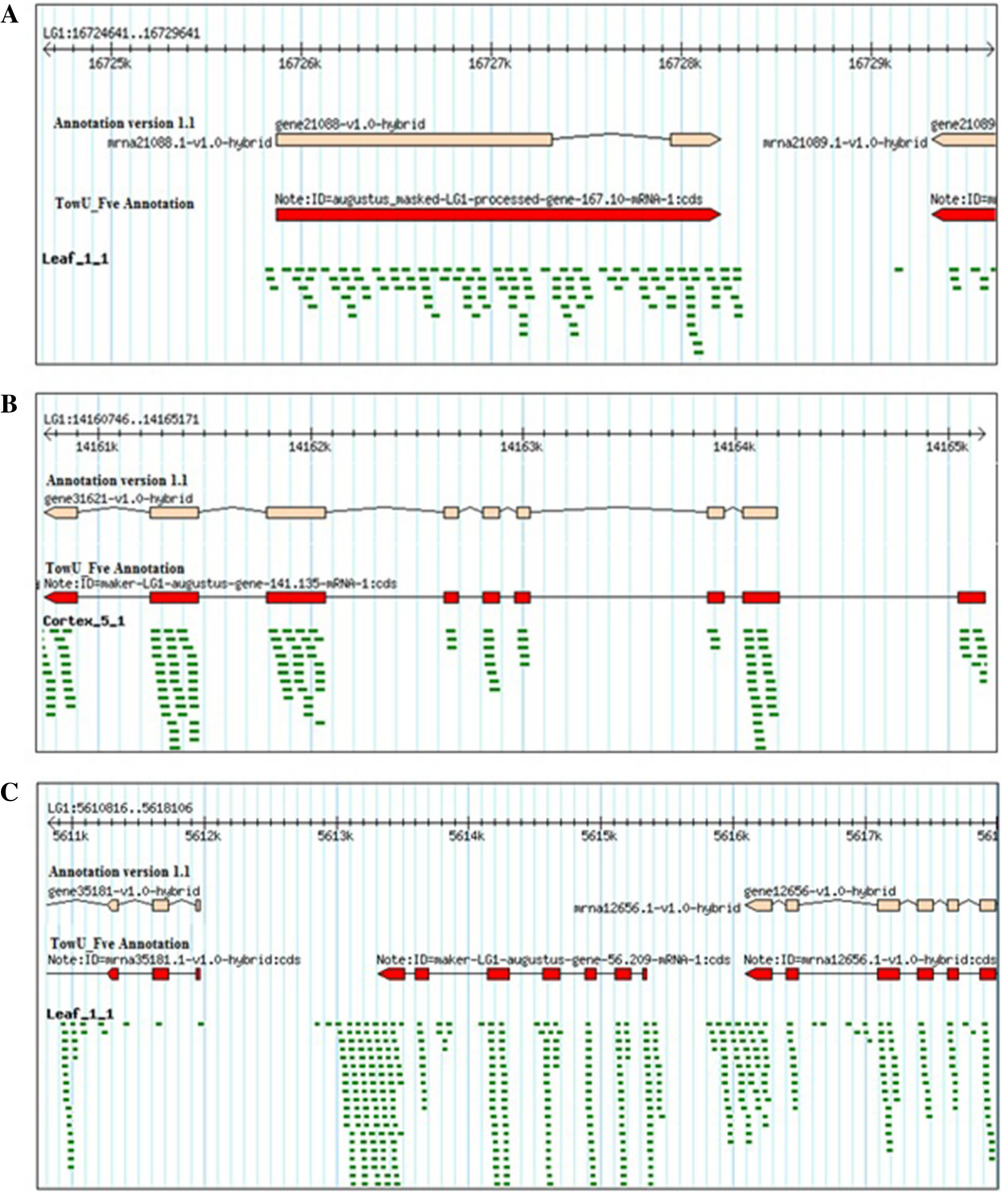
**Comparisons of version 1.1 annotation with the TowU_Fve annotation. (A)** The version1.1 (peach color) annotation shows two exons connected by an intron. However, leaf RNASeq reads align to the intronic region. The TowU_Fve annotation (red) merges two existing exons by including the intronic region. **(B)** The version1.1 (peach color) annotation is missing the last exon revealed by cortex tissue RNASeq reads alignments. The TowU_Fve annotation (red) shows the newly predicted gene structure with the addition of the distal exon. **(C)** The first generation annotation (peach color) shows an absence of a gene between 5613k and 5616k, while the aligned reads from leaf tissue revealed the existence of an expressed gene at that site. The TowU_Fve annotation (red) shows a newly predicted gene (an alpha/beta-hydrolase domain-containing protein) between gene35181 and gene12565.

The increased numbers of coding regions were discovered based on the RNA-Seq reads from different tissue libraries, as illustrated in Figure 3. For example, Figure 3A illustrates that the transcriptome data uncovered potential splice variation for gene21088, a putative receptor protein kinase, and re-annotation resulted in retention of a previously annotated intron. Figure 3B shows the addition of an exon to gene31621, a bZIP transcription factor. Figure 3C illustrates the discovery of a new gene, a putative hydrolase, absent from previous annotations.

**Figure 4:**
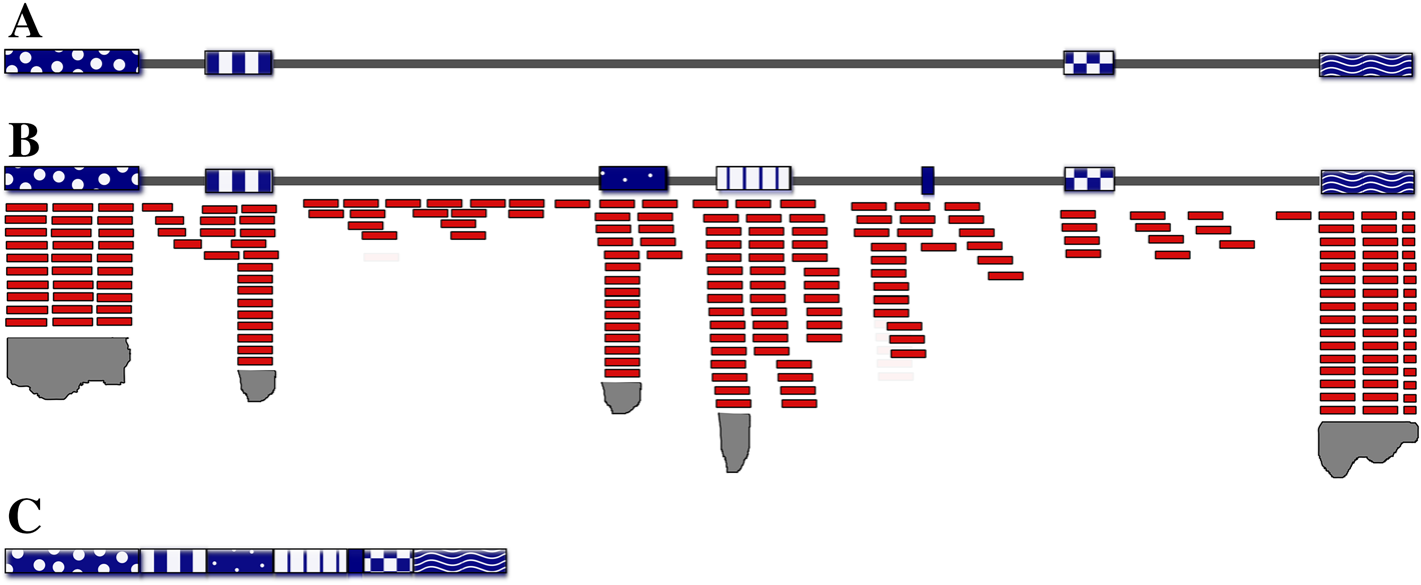
**cDNA sequences support the re-annotation of gene11268. (A)** *F. vesca* gene11268 annotation predicted by the first generation annotation at GDR. Colored boxes denote exons and gray lines denote introns. **(B)** Re-annotation of *F. vesca* gene11268 revealed the presence of additional exons. RNA-Seq reads from stage 7_8 anther are represented as red rectangles. Gray peaks below the red rectangles represent the abundance of additional reads beyond those shown. **(C)** The TowU_Fve predicted structure of *F. vesca* gene11268 after splicing is supported by the sequence of cDNA clones from YW5AF7 anther mRNA. Sequences of two such cDNA clones were identical and yielded the TowU_Fve predicted gene structure as shown.

We PCR amplified, cloned and sequenced the cDNA of gene11268 encoding a MADS box protein to confirm experimentally the TowU_Fve annotation. TowU_Fve predicts additional exons in the second intron based on the RNA-seq data (Figure 4A,B). Figure 4C illustrates the amplified coding region found by sequencing two independent cDNA clones, which contained the additional TowU_Fve predicted exons.

Because the RNA-seq data was obtained from the inbred line YW5AF7, which is different from Hawaii4×4 on which the prior genome assembly was based, there remains some possibility that TowU_Fve predictions differ from the prior annotations because of genome sequence differences between the two lines. Nevertheless, TowU_Fve represents a substantial improvement over previous annotations and annotation differences due to sequence differences between YW5AF7 and Hawaii4×4 may potentially underlie interesting phenotypic differences between these two lines.

### Functional annotation of new gene models

Orthologous relationships between the new gene models predicted by the TowU_Fve annotation and other plants were evaluated by Blast2GO against the NCBI non-redundant protein database [15-17]. The goals of the GO analysis were to obtain support for the plant origin of the newly identified genes and to acquire information as to the function of these genes. The Blast2GO analysis showed that about 70% of the new gene models were found to have sequence homologies to plant proteins in GenBank (at e = 10^−5^) and could be assigned GO functions, or were found to contain a known protein domain using InterProScan. About 30% of the new gene models did not have any significant Blast hits or InterProScan identified domains.

## Conclusions

The *F. vesca* genome was sequenced and subsequently released in 2010, along with a first generation annotation (version 1.0) [5], that was subsequently replaced by version 1.1. Recently published deep transcriptome sequencing has shown that these previous versions of annotations were not completely accurate, as might be expected given that they were mainly derived from *ab initio* predictions combined with mapping high confidence ESTs and mapping of gene deserts from transposable elements. Accurate and detailed genome annotation for diploid strawberry would be a valuable resource for *Fragaria* and the entire *Rosaceae* family. Seventy percent of the 2,286 new gene models identified by TowU_Fve have homologs in other plant species and/or have known GO ontologies. The remaining 30% potentially encode proteins of special interest to the *Fragaria* research community. The revised annotation, based on transcriptome sequences from a large number of different tissue samples, represents an important milestone in improving the accuracy of the diploid strawberry genome annotation. This improved genome annotation, TowU_Fve, provides a valuable resource for comparative and functional studies in flowering plants.

## Methods

### RNA-Seq Data

Tissue collection from the YW5AF7 cultivar of *Fragaria vesca* [3], RNA extraction, and sequencing were previously described in detail [2]. Briefly, plants were grown in growth chambers with 12 hours light at 25°C and 12 hours dark at 20°C. Samples were manually dissected from 25 different tissues (listed in Table 1) with two biological replicates for each tissue. cDNAs resulting from reverse transcription of RNA extracted from each of the 50 samples were sequenced on the Illumina HiSeq2000 platform using single-end chemistry with read lengths of 51 bp [2].

### *De novo* assembly and PASA alignments

More than 600 million single end reads were assembled using a two-step *de novo* assembly pipeline, Figure 1A. Sequence reads of the replicates were first merged into one library, and then merged libraries were assembled using Trinity [18]. This step generated redundant transcripts and transcripts that do not align to the genome. In the second step of the *de novo* assembly pipeline these issues were resolved using the Genomic Mapping and Alignment Program for mRNA and EST Sequences (GMAP) [14] within Program to Assemble Spliced Alignments (PASA) [19]. All *de novo* assembled transcripts from the first step were aligned to the *F. vesca* genome version1.1. The resulting alignments were then used as one of the inputs of the re-annotation pipeline. All transcripts not aligning to the genome were thereby discarded.

### Reference based assembly

The bioinformatics pipeline for the reference based assemblies of the transcriptome data is shown in Figure 1B. The first step was to align all reads from the 50 RNA-Seq libraries to the *F. vesca* genome version1.1 by passing each of the RNA-Seq samples through TopHat [20], resulting in 50 BAM files. TopHat aligns RNA-Seq reads to the reference genome in order to identify exon-exon splice junctions. It is built on the ultrafast short read mapping program Bowtie [20,21]. The BAM files from sample replicates were then merged using the “merge” command within SAMtools [22] reducing the number of BAM files to 25. All 25 BAM files were then sorted using the SAMtools “sort” command. The final step was to pass each of the 25 sorted BAMs to Cufflinks [23-25] to generate assemblies. Cufflinks assembles transcripts, estimates their abundances, and tests for differential expression and regulation in RNA-Seq samples [23-25], and uses the alignment generated by TopHat and GeneMark gene models to assemble the reference based transcripts.

### Training *ab initio* gene finding tools (GeneMark, Augustus, SNAP)

GeneMark models were built by training the GeneMark tool using the *F. vesca* genome version1.1. Augustus pre-trained datasets of *A. thaliana* and *S. lycopersicum* (tomato) were used along with the following: GeneMark models, *de novo* assembled transcripts, reference-guided assemblies, GDR annotation, and reference sequence proteins to run the first round of the MAKER annotation pipeline. The gene models, generated from the first round of the MAKER annotation pipeline were then used to train the SNAP tool.

### MAKER annotation pipeline

All *de novo* assembly steps were executed on the Data Intensive Academic Grid (DIAG), a shared computational cloud that is available for academic and non-profit institutions for performing bioinformatics analyses http://diagcomputing.org/about/investigators.php.

All MAKER runs were executed on the iPlantCollaborative (http://www.iplantcollaborative.org/) cloud infrastructure service platform. We used the virtual machine instance emi-490420DC size c1.xlarge (16 CPUs, 16 GB memory and 50 GB disk) to run the MAKER annotation pipeline.

The following tools were installed on a personal computer 64-Bit CentOS 6. Bowtie2 (http://bowtie-bio.sourceforge.net/bowtie2/, used to align the RNA-Seq libraries to both the *F. vesca* genome and GDR predictions); PASA (http://pasa.sourceforge.net/, used to align *de novo* assembled transcripts to the *F. vesca* genome); and Cufflinks (http://cole-trapnell-lab.github.io/cufflinks/, used perform the reference-guided assembly).

The MAKER annotation pipeline was utilized to automatically synthesize the following input data for a final run into gene annotations with evidence-based quality values. The data used as input into the MAKER pipeline shown in (Figure 2) are: *de novo* assemblies, reference-guided assemblies, reference sequence proteins, Augustus trained datasets, SNAP trained models, GeneMark models and the GDR annotation.

### Experimental verification of gene11268

Stage 12 anthers were dissected from YW5AF7 flowers and total RNA was extracted using the RNeasy Plant Mini Kit (Qiagen, www.qiagen.com) in conjunction with RNase-free DNase (Qiagen). PolyA selection and cDNA synthesis were conducted using the iScript cDNA Synthesis Kit (BioRad).

The full length cDNA of gene11268 (621 bp; spanning exon 1 to 7) was PCR amplified using Phusion (NEB) polymerase with YW5AF7 stage 12 anther cDNA as template. PCR primer sequences were: F: 5’ ATG GGG AGG GGT AAG ATT GAG 3’ and R: 5’ TTA CAT TAT GTC GTG GAG ATT GGG CTG 3’. PCR conditions were as follows: 98°C 30 s, 98°C 10 s, 57°C 30 s, 72°C 30 s, repeat steps 2-4 34 times, 72°C 10 min. The resulting fragment was cloned into pCR8/GW/TOPO using a TA cloning kit (Invitrogen). Plasmid DNA from two such cDNA clones was commercially Sanger sequenced using the insertion-flanking GW1 and GW2 primers (Invitrogen).

### Availability of supporting data

The TowU_Fve annotation files have been deposited at the GDR (http://www.rosaceae.org) for public release through their web portal. They can also be found at the Strawberry Genomic Resources database (SGR) (http://bioinformatics.towson.edu/strawberry/TowU_Fve_Annotation.aspx). RNA-Seq data are available at BioProject Accession number: PRJNA187983 (http://www.ncbi.nlm.nih.gov/bioproject/?term=PRJNA187983) and ArrayExpress Accession Number: SRR674059 (http://www.ebi.ac.uk/ena/data/view/SRR674059).
